# Phage-mediated horizontal gene transfer and its implications for the human gut microbiome

**DOI:** 10.1093/gastro/goac012

**Published:** 2022-04-13

**Authors:** Tatiana Borodovich, Andrey N Shkoporov, R Paul Ross, Colin Hill

**Affiliations:** 1 APC Microbiome Ireland, University College Cork, Cork, Ireland; 2 School of Microbiology, University College Cork, Cork, Ireland

**Keywords:** gut phageome, horizontal gene transfer, gene transduction, phage-mediated gene transfer

## Abstract

Horizontal gene transfer (HGT) in the microbiome has profound consequences for human health and disease. The spread of antibiotic resistance genes, virulence, and pathogenicity determinants predominantly occurs by way of HGT. Evidence exists of extensive horizontal transfer in the human gut microbiome. Phage transduction is a type of HGT event in which a bacteriophage transfers non-viral DNA from one bacterial host cell to another. The abundance of tailed bacteriophages in the human gut suggests that transduction could act as a significant mode of HGT in the gut microbiome. Here we review in detail the known mechanisms of phage-mediated HGT, namely specialized and generalized transduction, lateral transduction, gene-transfer agents, and molecular piracy, as well as methods used to detect phage-mediated HGT, and discuss its potential implications for the human gut microbiome.

## Introduction

The human gut microbiome is a complex community with a vast network of microbe–host interactions. The ability to change and adapt to acute events is essential to maintain long-term stability. The microbial community, with its short generation times and intense selective pressure, evolves at a speed unimaginable for multicellular organisms, and can therefore respond more quickly than its host to changing environmental conditions [1]. This is further enhanced by the ability of the microbiome to exchange genetic material that ensures swift access to an extensive bacterial pan-genome [2].

Genetic information is typically transferred vertically from parent to offspring and is subject to change through mutation. This is how a phylogenetic tree depicts evolution. Any other movement of genetic information is referred to as horizontal gene transfer (HGT). In recent years it has become increasingly clear that without taking HGT into account, it is impossible to describe the evolution of microbial communities [3], especially those as complex as the human gut microbiome. A phylogenetic web or network, rather than a tree, has been proposed to comprehensively depict these evolutionary relationships [1, 4]. Moreover, it has been shown that a dense network of HGT connects members of the human microbiome [2] and evidence of extensive horizontal transfer has been found in the human gut microbiome in particular [5].

HGT within the microbiome has potential consequences for human health and disease. The spread of antibiotic resistance genes happens mainly by way of HGT [6]. Virulence factors that determine a strain’s pathogenicity can also be transferred horizontally [7], as can genes involved in metabolic functions [8], including the catabolism of certain sugars [9].

Several types of HGT events are well known in bacteria; these include transformation, conjugation, and transduction, as well as nanotube contact and vesicle-mediated transfer. Transformation is the uptake of free extracellular DNA by a competent bacterial cell (any cell that possesses the ability to capture, “ingest,” and incorporate free DNA into its genome). Conjugation describes the transfer of genetic material (typically but not always plasmids) between two bacteria through cell-to-cell contact, using specialized pili or direct adhesion. Another mode of HGT that also involves cell-to-cell contact and nanotube structures has been shown to facilitate transfer of non-conjugative plasmids in *Bacillus subtilis* [10]. DNA transfer in membrane vesicles has been described in the marine environment [11, 12], including “serial transfer” that enables the recipient to produce identical DNA-transporting membrane vesicles [13, 14]. This review focuses on transduction, the process in which a bacteriophage particle transfers non-viral DNA from one bacterial host cell to another. This occurs when bacterial DNA gets packaged inside some of the viral particles either in place of or together with bacteriophage DNA.

## A brief overview of phage replication and virion assembly

The number of viruses in the human gut virome is estimated to be >10^12^ [15] and the majority of these viruses are tailed bacteriophages (phages). This makes tailed phages the most abundant gene-transfer particles in the human gut microbiome. They are also perfectly suited for the task: all tailed bacteriophages (order *Caudovirales*) have double-stranded DNA (dsDNA) genomes with a virion structure optimized for carrying dsDNA. We can confidently predict that bacteriophages play a significant role in gene exchange in the human gut.

**Figure 1.**
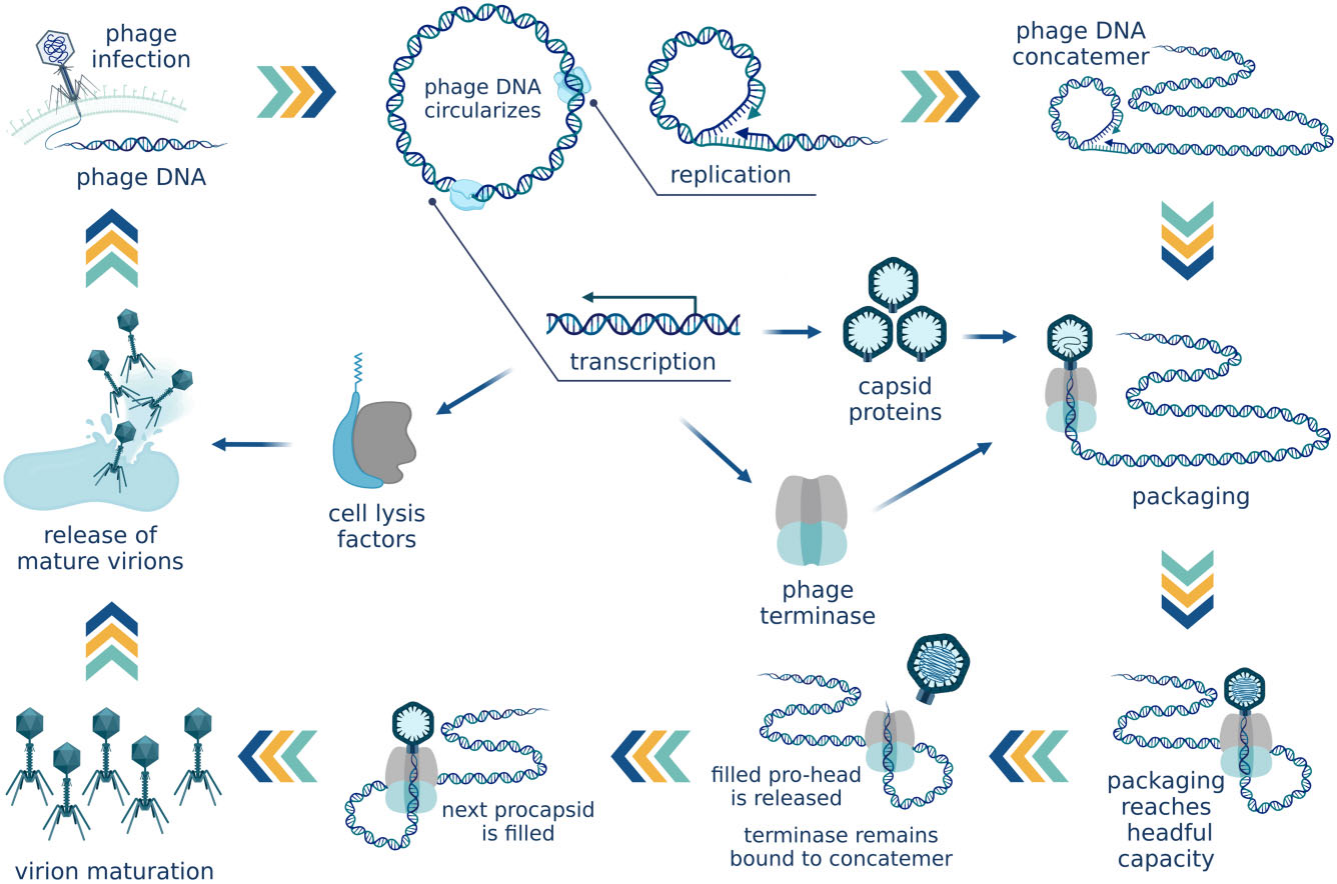
Phage reproduction. Rolling-circle replication produces long concatemers of the phage genome. Phage small terminase subunit (TerS) recognizes a *pac* site and initiates packaging. After one virion is filled with DNA, it disengages from the terminase complex, which remains attached to the DNA concatemer and binds to the next virion. (Created with BioRender.com.)

The normal propagation cycle of bacteriophages results in the production of viable virions that contain a single copy of the phage genome (Figure 1). Viable virions, or infectious particles, can infect and kill their host bacteria, producing phage progeny. The phage binds to the surface of the bacterial cell and injects its DNA into the cytoplasm. Lytic, or virulent, phages start propagating immediately and the progeny is released into the environment through cell lysis. Lysogenic (temperate) phages can also initiate the lytic cycle on entry into the cell. Alternatively, their genome can integrate into the host chromosome at a specific attachment (*att*) site. The integrated phage genome (prophage) replicates together with the host chromosome and is transferred vertically from the initial infected cell to its progeny through cell division. In an SOS response to DNA damage, the prophage excises from the host genome and enters the lytic cycle.

In the process of phage propagation, the phage genome, either having entered the cytoplasm from the infecting virion or having been freshly excised from the host DNA in response to inducing factors, circularizes and starts replicating. The first copies are produced through theta-replication, which ensures the generation of high-fidelity genome copies. Each of these copies then becomes a matrix template for rolling-circle replication that produces long covalent end-to-end polymers of the phage genome called concatemers.

Meanwhile, the translation of phage genes produces all the proteins required for the formation of viable phage particles. These include structural “head” and “tail” proteins, scaffolding and chaperone proteins that assist the correct assembly of the capsids, regulatory proteins, and the phage terminase whose function is to govern the packaging of phage genomes inside the phage heads.

The phage terminase complex consists of two subunits. The large terminase subunit (TerL) performs most of the mechanical work. It possesses endonuclease activity to cut the DNA, a complex packaging motor that translocates the DNA into the procapsid, and an ATPase domain that generates the energy used by the packaging motor. The small terminase subunit (TerS) performs regulatory functions. TerS is responsible for the packaging specificity of the terminase complex; it contains a DNA-binding domain that recognizes a specific tag sequence in the phage genome that labels it for cleavage and packaging. Having translocated one copy of the genome into the procapsid, the large subunit makes a second cut. The terminase complex remains bound to the free end of the concatemer, docks on another empty prohead, and continues packaging further headfuls of phage DNA while the filled capsid disengages and becomes ready for tail maturation [16]. Several types of phage terminases determine different strategies to ensure packaging specificity.

Headful packaging, the mechanism used by *pac*-type terminases (also called headful terminases), recognizes a single site in the phage genome called the *pac* site. When the small subunit of the terminase complex recognizes the *pac* sequence, the large subunit proceeds to package the DNA into the procapsid until the capacity of the phage head is reached, determining the site of the second cleavage by procapsid volume rather than a specific DNA sequence. Having packaged a full “headful,” which typically amounts to 102%–110% of the full phage genome size, the large subunit severs the packaged DNA from the rest of the concatemer [17]. This terminal redundancy created by packaging slightly more than a full-length bacteriophage genome becomes useful when the phage injects its DNA into the target cell where, once in the cytoplasm, the DNA will use its matching ends to circularize through recombination [18]. Among the phages, *Salmonella* phage P22 and *Escherichia coli* phages P1 and T4 are use this mechanism [19].

*cos*-Type terminases recognize two *cos* sites on the phage genome. The first *cos* site is required to initiate packaging and the second identical sequence acts as a signal for the terminase complex to introduce a second cut that separates the packaged DNA from the rest of the concatemer. As the DNA is not merely carried within the capsid but is itself an important part of the capsid structure, packaging of exactly the right length of DNA is crucial for virion assembly and maturation [16]. For this reason the terminase complex only becomes capable of recognizing the second *cos* sequence and making the cut once the procapsid is filled nearly to its capacity [16]. When cutting the DNA, *cos*-type terminases introduce two staggered cuts, first on one DNA strand and then the other, generating complementary, sticky ends used for circularization (cohesive ends). Bacteriophage lambda is the best studied model for *cos*-type phages [20]. A similar mechanism is used by phages with direct terminal repeats. After the terminase introduces staggered nicks in the phage DNA, the 3' end is extended to form identical blunt ends, as seen in *E. coli* phage T3 and *B. subtilis* phage SPO1 [21, 22].

Some bacteriophages, such as *B. subtilis* phage Φ29, differ not only in their packaging strategy but also in their mode of replication. They use protein-primed replication and do not produce concatemers. The terminase complex, which lacks the small subunit and endonuclease activity in the large subunit, binds to the terminal protein attached to each viral genome monomer and packages the genome copy [16].

Another notable strategy in the context of HGT belongs to *E. coli* bacteriophage Mu that replicates by transposition. Similarly to Φ29, its packaging substrates are genome monomers. On entry into the cell, the phage integrates randomly into the host chromosome and, when induced, produces multiple copies of its genome that re-integrate into random locations in the host genome. As a result the genome of phage Mu is always flanked by random fragments of host DNA [23]. Notably, the induction of phage Mu is low-frequency and spontaneous; no physical or chemical treatment is known to trigger lytic development of wild-type Mu [24].

As the human gut microbiome is a rich and diverse microbial community, it is likely to contain examples of all of the above strategies to ensure packaging specificity. Yet none of the mechanisms can completely exclude mispackaging and encapsidation of host DNA, and mechanisms of HGT by phage are even more diverse than the systems working against erroneous packaging.

## Mechanisms of phage HGT

### Specialized transduction

**Figure 2.**
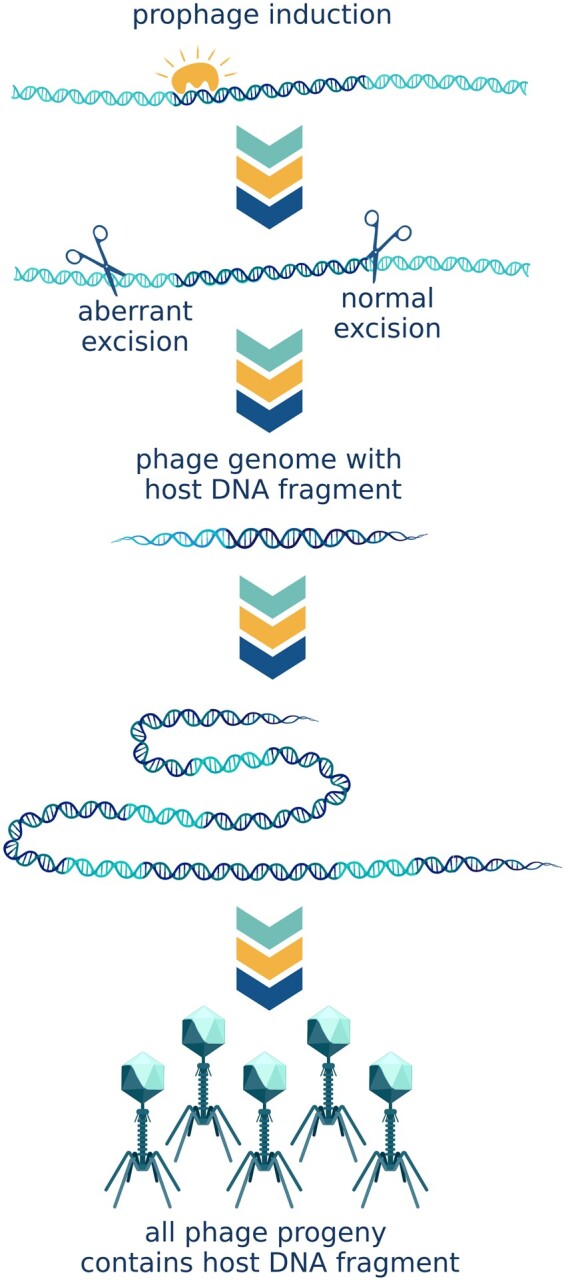
Specialized transduction. As a result of aberrant excision, adjacent host genes are cut out of the chromosome together with the phage genome. Host DNA becomes part of the circularized phage genome and is replicated. As a result, all the virions produced by the cell will carry a fragment of host DNA. (Created with BioRender.com.)

Specialized transduction can only be carried out by temperate phages and is normally restricted to those genes adjacent to the *att* site of the prophage. The induced prophage will occasionally excise imprecisely, cutting out adjacent host genes together with the phage genome. This host DNA becomes part of the circularized phage genome and is replicated. As a result of this aberrant excision event, all the virions produced by the cell will carry a fragment of host DNA (Figure 2). However, imprecise prophage excision is a rare event. In phage lambda for example, a transducing particle is produced by ∼10^4^ virions [25], while the reported rate of successful transduction is ∼1 in 10^6^ [26], which indicates that only one in ∼100 transducing particles leads to a completed transduction event.

Bacteriophage lambda, a classic example of specialized transducing phage, integrates into the genome of its host, *E. coli*, between the galactose metabolism and biotin biosynthesis operons. The phage can consequently transfer these genes, conferring a fitness advantage in certain environments. Likewise, in the gut microbiome, horizontal transfer of such valuable metabolic functions could facilitate adaptive changes in response to dietary alterations. Temperate bacteriophages constitute, from various estimates, 20%–50% of human gut phages [27]. Such a high prevalence of temperate phage supports the likelihood of significant levels of specialized transduction in the human gut.

### Generalized transduction

**Figure 3.**
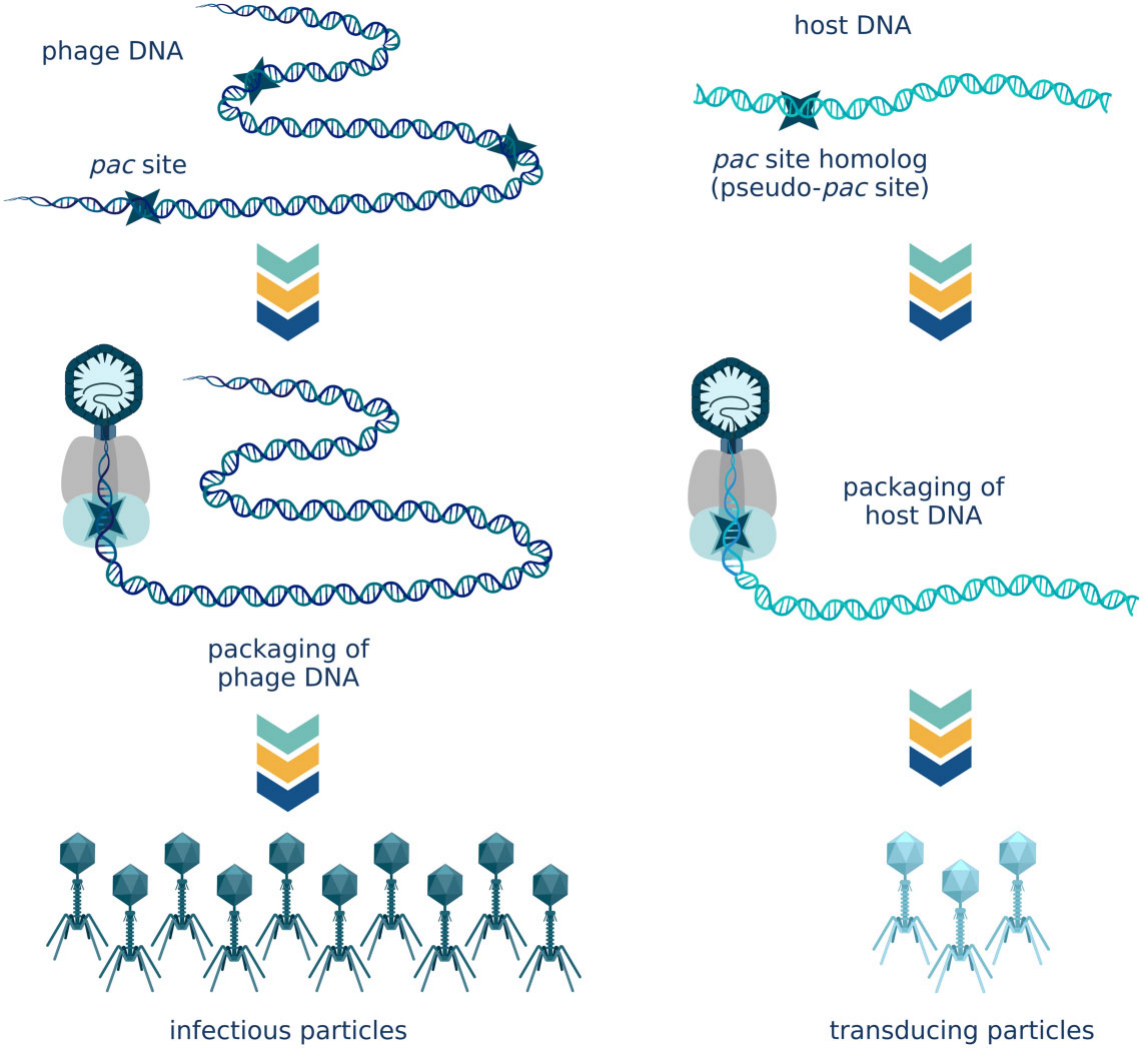
Generalized transduction. Instead of recognizing a bona fide *pac* site, the terminase complex recognizes a *pac* site homolog present in the host chromosome and proceeds to encapsidate phage-sized fragments of host genome until it disengages from the chromosome. (Created with BioRender.com.)

In generalized transduction, instead of recognizing a bona fide *pac* site, the terminase complex recognizes a *pac* site homolog present in the host chromosome and introduces a double-stranded break in its vicinity. Once the terminase machinery has encapsidated a “headful” of host DNA, a second break is introduced in the DNA. The filled virion is ready for tail maturation and detaches from the terminase complex. The terminase complex attaches to the free end of the bacterial chromosome and proceeds to encapsidate phage-sized fragments of host genome until it disengages from the chromosome [16] (Figure 3). The farther away from the initial pseudo-*pac* site, the higher the likelihood of the terminase complex detaching from the chromosome, and so transduction frequencies steadily decrease with increasing distance [25].

Generalized transduction is almost exclusively the domain of *pac* phages because headful (*pac*-type) terminases only require one *pac* site homolog in the host chromosome to package host DNA fragments into phage particles. For *cos* phages to engage in generalized transduction, the host genome would have to contain not one, but two *cos* site homologs located at an appropriate distance from one another [16]. Even though constrained by the location of host pseudo-*pac* sites, generalized transduction is unpredictable and thus inevitably adds yet another layer of complexity to HGT in the human gut microbiome. Generalized transduction is a low-frequency event. Only 1%–6% of virions contain host DNA [25, 28–30]. Of these transducing particles, 10%–15% are shown to inject their DNA into the host cells [31]. The number of complete transductions will be one to three orders of magnitude lower, depending on whether the recipient cells are recombinase-positive [31, 32]. Rare as it is, generalized transduction can facilitate significant adaptational changes in the human gut. If a transduction event involves genetic traits that dramatically increase fitness, it can initiate rapid spread of these traits in the population.

### Lateral transduction

**Figure 4.**
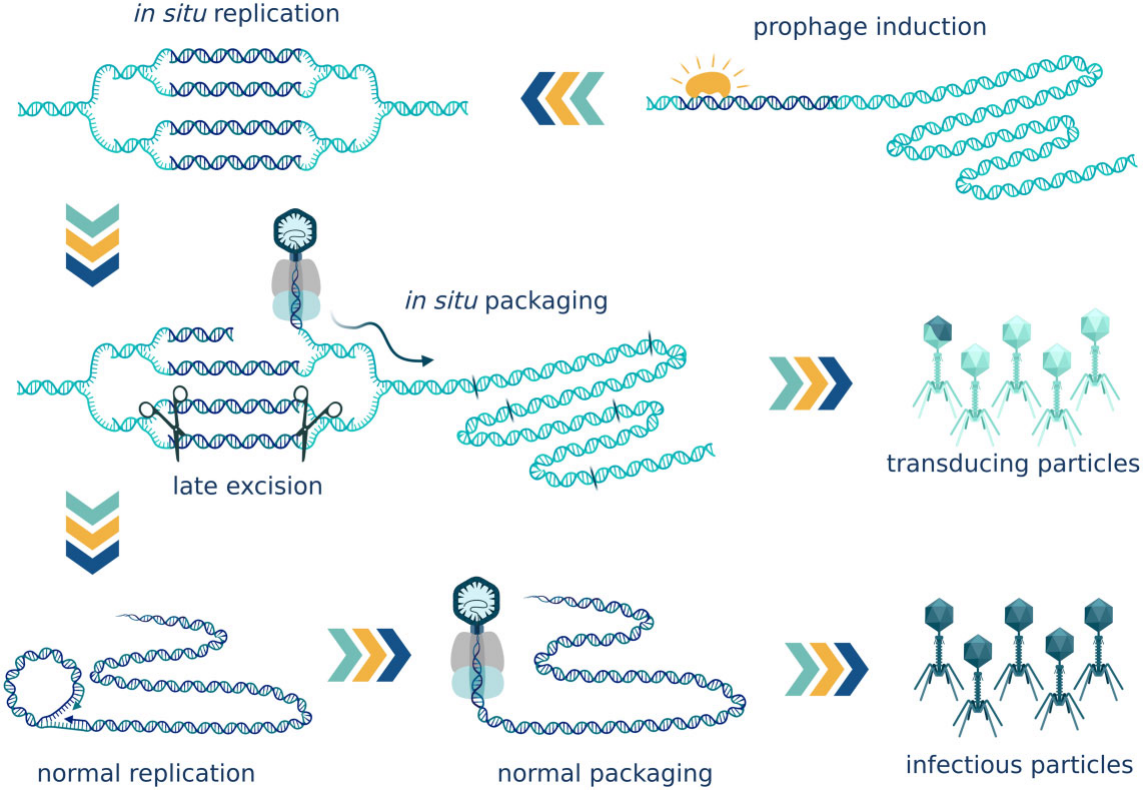
Lateral transduction. Lateral transducing phages start replicating while still integrated in the host chromosome. *In situ* replication creates multiple integrated phage genomes. Some of these genome copies later get excised and follow the typical cycle forming infectious particles. In those that stay integrated, the terminase complex initiates *in situ* packaging from the *pac* site near the middle of the phage genome and proceeds to package towards the host genome. The first virion contains a chimeric DNA molecule that is half phage and half bacterial. The terminase continues through the adjacent host chromosome efficiently packaging up to seven headfuls before the frequencies decrease. *In situ* packaging and the typical excision–replication–packaging cycle happen in parallel, producing both the transducing particles and the actual infectious virions. (Created with BioRender.com.)

Unlike generalized transduction, lateral transduction, such as observed in the prophages of *Staphylococcus aureus* [33] and *Salmonella* phage P22 [34], allows high-frequency host DNA transfer. Laterally transducing phages do not follow the typical excision–replication–packaging life cycle. Instead, they start replicating while still integrated in the host chromosome. *In situ* replication creates multiple integrated phage genomes. Some of these genome copies later get excised and follow the typical cycle forming infectious particles. In those genomes that stay integrated, the terminase complex recognizes the *pac* site located near the middle of the phage genome. It cuts the DNA at the *pac* site and proceeds to package without stopping at the junction between phage and host genomes. The resulting virion contains a chimeric DNA molecule that is half phage and half bacterial DNA. The terminase then continues through the adjacent host chromosome efficiently packing up to seven headfuls before the frequencies decrease to the rates of generalized transduction. In this manner, *in situ* packaging and the typical excision–replication–packaging cycle happen in parallel, producing both the transducing particles and the actual infectious virions [33] (Figure 4).

It has been observed that lateral transduction is a factor driving genome organization and the location of genes carrying useful adaptive traits. Virulence factors, pathogenicity determinants, resistance genes, and other genes responsible for fast adaptation to the change in conditions can be clustered in the regions of the chromosome adjacent to the phage integration sites in the direction of *in situ* packaging [33]. Lateral transduction was first described in the prophages of *S. aureus*, in which it creates a high-throughput gene-transfer channel promoting genome mobility and thus facilitating the swift adaptation of *S. aureus* to changing environments. The same need to constantly change and adjust to change is characteristic of the gut microbiome. Even though lateral transduction has so far only been observed in *Staphylococcus* and *Salmonella*, it is likely to occur in members of the gut microbiome, as well as in other environments.

### Molecular piracy

**Figure 5.**
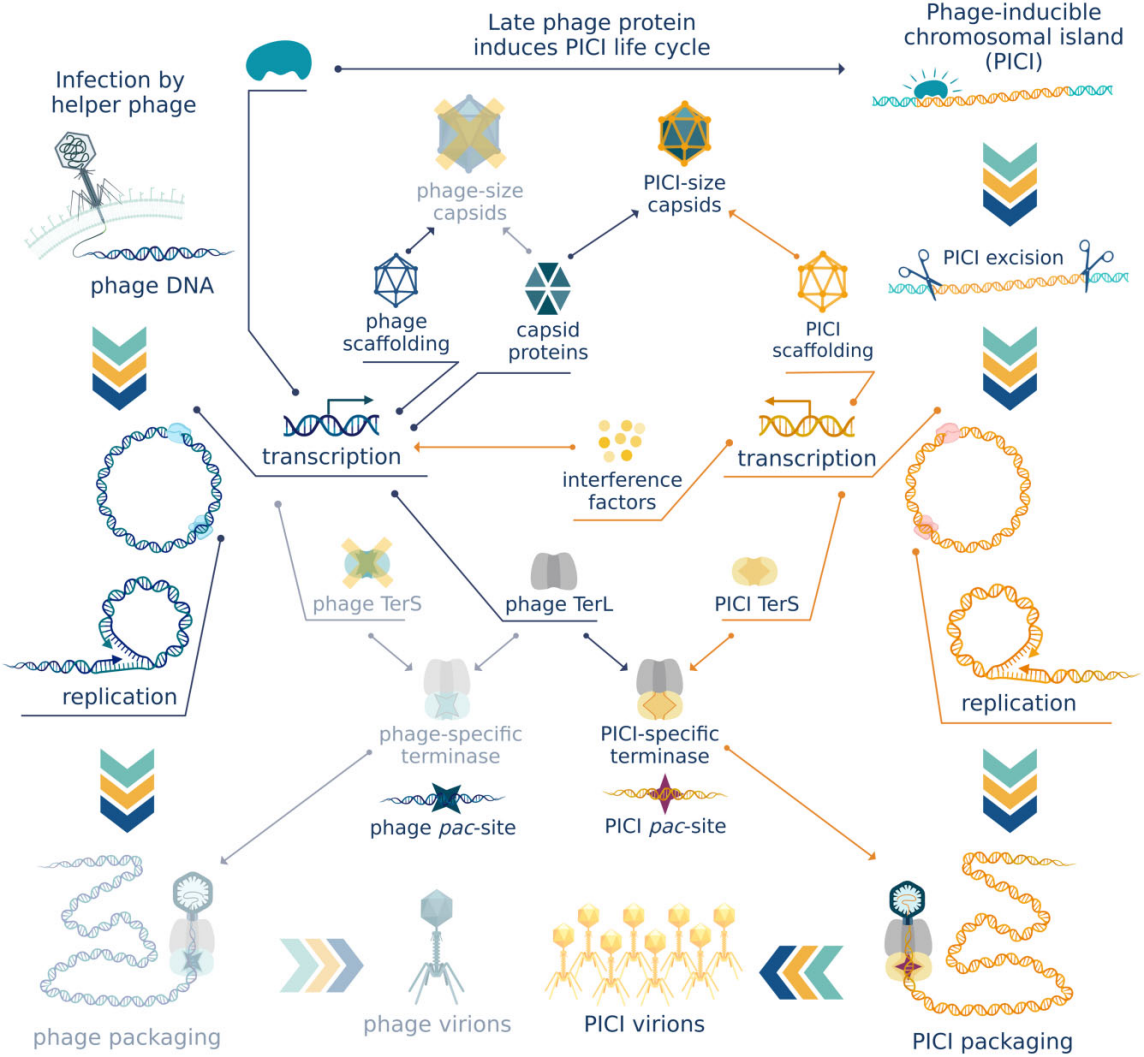
Phage-inducible chromosomal islands. Phage-inducible chromosomal islands (PICIs) are latent in the host genome until they are derepressed in the presence of an actively growing helper phage. PICIs hijack structural proteins and packaging machinery of the helper phage for transfer of PICI genes. PICI-specific small terminase subunit (TerS) forms a functional complex with phage large terminase subunit (TerL) while PICI interference inhibits the expression of phage TerS ensuring preferential packaging of PICI genes. PICI scaffolding proteins redirect capsid size to fit the smaller size of PICI genomes. (Created with BioRender.com.)

Another extraordinary mode of transduction was also first described in *S. aureus* [35]. Phage-inducible chromosomal islands (PICIs) are a family of phage satellites that exploit actively replicating phages for their dissemination. PICIs have a conserved genetic organization [36], but do not possess genes encoding phage structural proteins. Like prophages, PICIs are latent in the host genome until they are derepressed. However, unlike prophages, phage-inducible islands are not directly SOS-induced; instead, each responds only to a single-phage-encoded protein that serves as a PICI-specific derepressor [37]. The phage antirepressor protein disrupts the complex between the PICI master repressor and its binding site on the PICI genome [37], enabling the transcription of most of the PICI genes. Transcription of early genes is followed by PICI genome excision, circularization, and replication. The derepression mechanism ensures that PICIs only get induced in the presence of an actively propagating helper phage, whose capsids they hijack for transfer to a new host cell (Figure 5).

Some PICIs replace phage-encoded scaffolding proteins during the making of procapsids and redirect the capsid size to fit their considerably smaller genomes [38, 39], while other PICIs simply use normal-sized helper phage capsids, packaging more than one genome copy inside each virion [40]. Most known PICIs encode their own TerS but no TerL [36]. Two key features are characteristics of PICI-encoded TerS. First is the formation of a functional complex with the TerL encoded by the helper phage. The second is the recognition of the *pac* sequence of the PICI instead of that of the helper phage. TerS will guide the large subunit to package the chromosomal island genome while ignoring the helper phage genome entirely. To increase the probability of phage TerL forming a complex with PICI TerS, PICI-encoded repressor proteins target the transcription of phage-encoded TerS [41]. However, PICIs do not encode a TerS sequence. Instead, they contain a *pac* site recognizable by phage-encoded terminase-like PICI elements of *Enterococcus faecalis*, or a pair of *cos* sites located at appropriate distance from each other as observed in the chromosomal islands of *E. coli* [42]. Both features can also be present, as seen in the *S. aureus* PICI SaPIbov5 [43].

PICIs severely impair helper phage reproduction, ensuring that only functions that benefit the satellite are preserved [41, 44]. As a result, most of the produced virus-like particles (VLPs) contain a PICI genome. For instance, in the *E. faecalis* EfCIV583-Φ1 pirate-helper system, almost 10 PICI-carrying particles are produced for each bona fide helper phage virion [25, 45]. An even more extreme case is the *Vibrio cholerae* PICI-like element PLE1. When cells carrying this element are infected with its helper phage ICP1, no phage progeny are produced [46, 47].

Helper phages and their satellites undergo rapid co-evolution with phages developing resistance to the chromosomal islands and the PICIs in turn evolving to evade that resistance. Mutations give rise to allelic polymorphism in the genes encoding derepressor proteins for PICIs. It has been observed that *E. faecalis* phages that had interacted with PICI elements accumulated mutations in the gene acting as the inducer for the chromosomal island [48]. PICIs, in turn, mutate the master repressor binding site in a way that allows its complex with the master repressor to be disrupted by the new allelic variant of the derepressor protein [48]. *Vibriocholerae* bacteriophage ICP1 encodes its own CRISPR/Cas system against PICI-like elements that parasitize ICP1 and prevent it from producing progeny [46].

While PICIs are detrimental to helper phage, they can benefit their bacterial hosts, not only by reducing the number of infectious particles produced by their helper phage, but also by carrying genes that increase the host’s fitness. The discovery of the first-phage-inducible islands was prompted by studies of toxic shock syndrome toxin encoded by a PICI element [35]. Many more accessory genes that encode virulence and pathogenicity factors, resistance pathways [49], and other instruments of swift adaptation have been identified in PICIs. For instance, in *S. aureus*, PICIs determine animal host specificity [50]. Since their discovery in *S. aureus*, phage-inducible islands have proven to be ubiquitous among both Gram-positive and Gram-negative bacteria. Evidence points to known PICIs not sharing a common ancestor. Known PICI elements seem to belong to at least two distinct lineages, likely evolved on separate occasions from different prophage ancestors [42].

PICI elements have been described in human gut commensals such as *E. faecalis*. The presence of phage-inducible mobile elements in the genomes of human gut residents can impact the data we obtain from sequencing fecal viromes. For instance, an actively reproducing PICI element will produce high-coverage contigs that lack structural proteins typically used to identify a sequence as viral, yet contain other common markers of viral origin. This situation may further complicate our attempts to shed light on what we know as “viral dark matter.”

PICIs are known to use multiple phages as helpers [36]. Even a non-helper phage can be used by a PICI element for transfer by means of generalized transduction if the PICI genome is located in the proximity of a non-helper phage pseudo-*pac* site in the direction of packaging. Due to their ability to integrate into the host genome, a higher frequency of successful transduction events is observed as compared to the transfer of a random genome fragment [36].

As shown in *S. aureus* [36], chromosomal islands that encode their own TerS can mediate low-frequency generalized transduction. The PICI-encoded TerSwill recognizes the PICI *pac* site homologs and packages adjacent fragments of host DNA. Interestingly, PICI-mediated generalized transduction is not restricted to cases of active PICI reproduction. PICI TerS gene is part of operon I and is activated by the SOS response independently from PICI induction, even in the absence of an actively growing helper phage. This allows the chromosomal island to hijack any non-helper phage induced by the same SOS response for PICI-driven generalized transduction, so long as that phage uses *pac*-type packaging and possesses a TerL that can form a functional complex with PICI-encoded TerS. Predictably, this phenomenon drives genome organization, with pathogenicity and resistance genes clustering near to PICI pseudo-*pac* sites [51].

It has been shown that PICIs of *S. aureus* can be transferred to other *Staphylococcus* species and even to *Listeria monocytogenes* [52, 53]. The helper phage does not need to be able to reproduce in the recipient strain; it need only attach to the cell surface and inject its DNA into the cytoplasm. In this case, intergeneric transfer is possible because the helper phage uses non-specific teichoic acids for binding to its host cell and many Gram-positive genera share the structure of these polymers [54, 55]. Once in the cytoplasm, the PICI element will be able to integrate into the host genome if there is a serviceable *att* site present in the genome [52].

A somewhat similar yet unique case of molecular piracy is the *E. coli* integrative plasmid P4 (also known as satellite phage P4) [56, 57]. Similarly to PICIs, this element uses structural proteins of its helper phage P2 for transfer, redirecting the capsid size to fit its smaller genome [58, 59]. P4 is induced in the presence of its helper phage and, interestingly, can induce the helper [60]. In the absence of its helper, phage P4 can reside quiescently integrated in the host genome. Alternatively, P4 can excise and persist as a multiple copy plasmid [61]. P4 converts to its plasmid state in only ∼1% of cases [61]. Sequences similar to P4 are widespread among *E. coli* strains [57]. A similar but unrelated integrative plasmid has also been described in archaea [62].

### Gene-transfer agents

**Figure 6.**
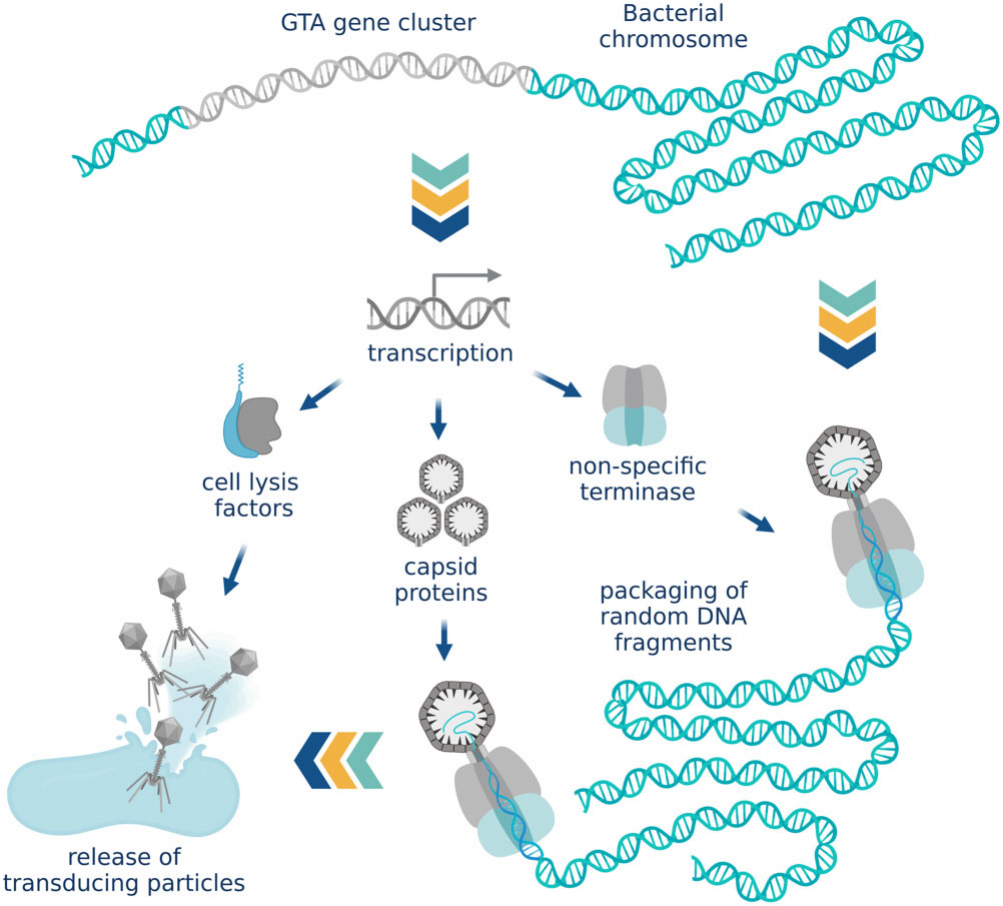
Gene-transfer agents. Gene-transfer agents (GTAs) are phage-like particles that encapsidate random fragments of bacterial DNA and are released into the environment by cell lysis. The size of GTA particles is not sufficient to transfer the full cluster of genes that encodes them. (Created with BioRender.com.)

Whereas PICIs are “selfish” mobile elements with no structural genes that hijack helper phage capsids to spread in the population, gene-transfer agents (GTAs) are phage-like particles [63] that encapsidate random fragments of their “host” DNA and are released into the environment by cell lysis to facilitate adaptation and survival of the surrounding cells, but without transferring the GTA-encoding genes (Figure 6). With their typical headful capacity of 4–14 kb, GTAs are too small to carry their own full set of genes and GTAs also lack specificity in DNA packaging [64]. They are vehicles of genetic mobility rather than mobile genetic elements. Gene transfer by GTAs does not technically involve bacteriophages. However, practically speaking, GTAs are morphologically similar to tailed phages and are subsequently found in viral metagenomes [25].

GTA genes typically contain complete bacteriophage morphogenesis and lysis modules but lack the genes for replication, integration, and excision [28]. The expression of the GTA gene cluster is regulated by cellular systems and modulated by quorum sensing pathways [64, 65] rather than a phage-typical repressor mechanism. GTAs most likely use headful packaging strategy for encapsidating the genomic DNA but no specific *pac* sites have been identified [66].

Interestingly, it has been shown in *Rhodobacter capsulatus* that DNA carried by GTAs is only injected as far as the cell’s periplasm (the space between outer and inner membrane in Gram-negative bacteria) [63]. Further uptake of the DNA into the cell’s cytoplasm requires homologs of competence pathway proteins that are involved in DNA uptake via transformation [67]. The process is regulated by the same protein that controls GTA production [63].

Several unrelated families of GTAs have been identified in proteobacteria, spirochetes [64], and archaea [68]. In *B. subtilis*, GTA-like elements are produced by the defective resident phage PBSX [69]. Similarly to the GTAs, PBSX contains the full morphogenesis module but lacks the ability to replicate [70]. When induced, PBSX randomly packages 13-kb fragments of the host chromosome into VLPs that are released through cell lysis. The important distinction between PBSX GTA-like particles and bona fide GTAs is that PBSX particles do not seem to produce any detectable transductants, instead acting similarly to bacteriocins by inhibiting the growth of PBSX-negative cells and thus creating selective pressure for the PBSX element [64].

Similar to lateral transduction, GTAs promote genome mobility and can potentially increase the adaptability of the gut microbial community.

### Fate of the transducing DNA

Unlike transformation and conjugation that can transfer DNA across large phylogenetic distances, transduction is constrained by the host range of the carrier phage particle. The transducing particles carrying cellular DNA are morphologically identical to the parent phage, including tail receptors. It will subsequently be able to attach to the cell surface of any strains or species that the original phage can attach to and can then inject the packaged DNA into the cytoplasm. However, the bacteriophage does not need to be able to successfully reproduce in the recipient cell for the transduction event to take place. In consequence, the bacteriophage’s potential taxonomic range for transduction may differ significantly from its host range as certain bacteriophages are known to adsorb on bacterial cells in which they are unable to reproduce [53]. Still, most transduction events appear to take place within a narrow taxonomic range restricted to closely related strains and species with highly similar genomes [71] and are further constrained by ecological barriers.

Once the transducing DNA enters the cell, it is either degraded by intracellular nucleases, lost in subsequent cell divisions, or gets incorporated into the host chromosome. In the case of the *E. coli* generalized transducing phage P1, <3% of the DNA from transducing particles is integrated into the recipient chromosome [31]. Only a fragment of the transducing DNA is typically integrated, usually significantly smaller than the entire phage genome-sized transducing DNA [31]. Between 10% and 15% of the DNA is degraded and recycled. The rest persists without replications or integration for at least several generations, becoming gradually diluted in the population.

Transducing DNA is integrated into the chromosome by homologous recombination, performed by RecA, a highly conserved DNA-repair protein. The process can only integrate DNA that has some degree of sequence identity to the recipient’s DNA, thus restricting transduction events to homologous regions. After the transducing DNA has integrated into the chromosome, there are fitness consequences to be considered. The integration itself appears to come at a cost. While the typical generation time of an *E. coli* culture is 30 min after a 30-min lag phase, the transductants take 180 min to double in number, meaning a lag of about three generation times. The same has been observed for *Salmonella* phage P22 transductants [72]. In consequence, the transductants can be outcompeted by faster-growing cells.

Genetic traits carried by the transducing DNA may also impact cell fitness. The acquired traits can be beneficial, neutral, or detrimental to the recipient. In case of the latter, the recipient cell’s progeny will be outcompeted and the acquired genes will be quickly lost in the population. Conversely, if the acquired trait dramatically increases fitness, it has the potential to rapidly spread throughout the microbial community. It has been shown that most horizontally acquired genes are neutral or nearly neutral [1] and that most completed transduction events introduce genes that are highly similar to those of the recipient [71]. Such duplicating transductions are believed to happen more often than internal gene duplication and provide the material for later adaptation, rather than directly introducing genetic innovations into the genome [71].

Another factor working against transductants is the concurring presence of intact infectious particles. Experimental studies of transduction are usually carried out under conditions that prevent the production of infectious particles and subsequent superinfection, which is not the case for naturally occurring transduction events. The cell “infected” with the transducing particle will have no bacteriophage-encoded immunity against superinfection. As the cells around it become infected with infectious particles and phage progeny is produced, the likelihood of the recipient cell getting infected and lysed becomes higher. Overall, the number of transducing particles is typically 10- to 1,000-fold higher than the number of completed transduction events [25, 31, 32].

## Finding evidence of phage HGT in gut metagenomes

### Reconstructing historical HGT events

Historical HGT events can be identified by observing phylogenetic incongruence; the genes that were acquired by horizontal transfer may display an evolutionary descent pattern different from that of the rest of the genome [3]. Where transduction events take place between closely related species or strains, close relation of donor and recipient will mask this phylogenetic evidence. The spatial distribution of the horizontally transferred genes in closely related lineages will be inconsistent with common ancestor patterns, and the order and composition of transferred gene clusters will often be indicative of HGT [3]. Distinguishing historical transduction events from other types of HGT poses a challenge. In the case of specialized transduction or phage morons (genes of cellular function encoded by a bacteriophage), these events can be detected by association of the acquired genes with prophage-related elements [71]. However, transduction events mediated by non-temperate phages or transduction events in which transducing DNA arrives unassociated with bacteriophage genome, as is the case for generalized transduction and GTAs, are essentially indistinguishable from other types of horizontal transfer.

### Recognizing ongoing phage-mediated HGT in the gut microbiome

It is possible to observe nascent transduction events in the gut by purifying VLPs from fecal samples, sequencing the packaged DNA, and looking for bacterial sequences in the resulting viral metagenome. In theory, any bacterial DNA found in the purified VLP metagenome would constitute evidence of ongoing transduction. However, this approach is constrained by both imperfections of purification methods [73, 74] and a lack of comprehensive knowledge of bacteriophage genomes that would allow precise identification of a sequence as being of viral or bacterial origin [15, 75]. Even with the most rigorous purification techniques (e.g. using CsCl gradient centrifugation), a certain level of “noise” can produce non-specific coverage signals [25]. Genuine transduction signals can be distinguished from non-specific noise when coverage patterns of bacterial contigs in the viral metagenome are compared to bacterial genome coverage patterns from single-phage preparations of transducing bacteriophages. This approach, termed “transductomics,” makes it possible to detect coverage patterns characteristic of a specific transduction mechanism in the overwhelming amount of sequencing data from a gut virome [25]. Unlike the historical approach, this method can be used to observe all ongoing potential gene transfer through phages, including incomplete HGT events that will not lead to retention of the acquired DNA, but does not make it possible to determine the transduction efficacy. For example, GTA-like particles produced by defective *B. subtilis* phage PBSX do not produce transductants, but they carry host DNA and will be identified as transducing particles using this approach [25].

## Discussion

Transduction frequencies are known to vary between habitats [71]. No reliable data exist on the frequency of transduction in human gut microbiome, but some findings point to the likelihood of high transduction frequencies. As observed by reconstruction of historical HGT events, both overall horizontal transfer [2] and transduction events [71] are enriched between human-associated bacteria as compared with bacteria from other environments. Moreover, it has been shown that prophages are induced during gastrointestinal transit [76], which would be expected to result in increased frequency of transduction.

Transductomics analysis suggests that 8.6% of bacterial contigs in murine gut metagenome display transduction signals, of which one-quarter have patterns inconsistent with any known mode of transduction but that are distinct from non-specific contamination or erroneous read mapping [25]. Such a high occurrence of unidentified transduction patterns illustrates that our understanding of phage-mediated gene transfer remains incomplete. An experimental gut-colonization study in mice suggests that evolution by phage HGT precedes and overshadows evolution by mutation in *E. coli* [77]. The same study demonstrates a multifaceted context for phage HGT events in gut microbiomes involving the interplay between environmental conditions, phage–host and phage–phage interactions, prophage immunity, and metabolic adaptations.

It is highly likely that phage-mediated horizontal transfer in the gut microbiome is involved in health and disease outcomes in humans, but no reliable data exist to confirm or refute this hypothesis. Gut microbiome and virome alterations have been linked to the immune system [78, 79], mental health [80–83], obesity [84–86], type 2 diabetes [84, 85], and potentially coronary heart disease [87]. Specific mechanisms of gut microbiome involvement have not yet been determined. It is possible that a balance of certain metabolic functions or virulence factors underlies some of the above correlations, and the genes determining such functions might be mobilized through phage-mediated transfer. Transfer of antimicrobial resistance genes by phages remains a subject of debate [75, 88–90], showcasing the need for further research and better understanding of phage-mediated gene transfer, as well as the need to develop more reliable methodology to analyse viral metagenomes.

We believe that understanding phage-mediated gene transfer is crucial in interpreting complex ecological interactions within the human gut microbiome and is therefore worthy of further investigation.

## Authors’ Contributions

Conceptualization: A.N.S. and T.B.; Writing—original draft: T.B.; Writing—revision and editing: T.B., A.N.S., R.P.R., and C.H.; funding acquisition: A.N.S., R.P.R., and C.H. The authors read and approved the final manuscript.

## Funding

This publication has emanated from financial support from Science Foundation Ireland [grant number SFI/12/RC/2273_P2] and Wellcome Trust under a Wellcome Trust Research Career Development Fellowship [220646/Z/20/Z] (A.N.S.). This research was funded in whole, or in part, by the Wellcome Trust [220646/Z/20/Z]. For the purpose of open access, the authors have applied a CC BY public copyright licence to any Author Accepted Manuscript version arising from this submission.
